# West Nile Virus in Birds, Argentina

**DOI:** 10.3201/eid1404.071257

**Published:** 2008-04

**Authors:** Luis Adrián Diaz, Nicholas Komar, Andres Visintin, María Julia Dantur Juri, Marina Stein, Rebeca Lobo Allende, Lorena Spinsanti, Brenda Konigheim, Javier Aguilar, Magdalena Laurito, Walter Almirón, Marta Contigiani

**Affiliations:** * Universidad Nacional de Córdoba, Córdoba City, Argentina; † Centers for Disease Control and Prevention, Fort Collins, Colorado, USA; ‡ Universidad Nacional de Tucumán, Tucumán, Argentina; § Universidad Nacional del Noreste, Chaco, Argentina

**Keywords:** West Nile virus, birds, Argentina, flavivirus, St. Louis encephalitis virus, serology, arboviruses, letter

## Abstract

West Nile Virus in Birds, Argentina

**To the Editor:** West Nile virus (WNV), genus *Flavivirus*, family *Flaviviridae* has been rapidly dispersing through the Americas since its introduction in 1999 in New York (*1*). By 2004, serologic studies detected WNV-specific antibodies in birds and horses from Canada to northern South America (*2**–**4*). The first report of WNV activity in the Southern Cone of South America surfaced in April 2006, when 3 horses died in Argentina (*5*). However, established transmission foci in Argentina are unknown. We report evidence for the introduction and establishment of WNV in Argentina as early as January 2005.

Serum samples from free-ranging birds were collected from 5 locations in Argentina and screened for generic flavivirus antibodies by using a blocking ELISA with monoclonal antibody 6B6C-1 (*6*). Positive serum specimens were further characterized by plaque-reduction neutralization test (PRNT). We identified the etiologic agent responsible for the previous flavivirus infection by using the following criteria: 80% neutralization of reference virus (WNV NY99-4132 or an Argentinean strain of St. Louis encephalitis virus [SLEV CbaAr4005]) in serum diluted at least 1:40 and 4-fold greater titer compared with the other virus.

Overall, 474 (25.6%) of 1,845 serum specimens from 117 bird species collected from January to June 2006 tested positive when using the blocking ELISA; 30% inhibition was the threshold for a positive test. SLEV infections were confirmed in 105 birds by PRNT; WNV infections were confirmed in 43 birds. Anti-WNV antibody titers ranged from 40 to 2,560 in birds collected as early as January 2005 in Córdoba City and as late as June 2006 in Mar Chiquita (Table). Recent WNV activity was indicated by seroconversion in 3 banded rufous hornero in Córdoba City between January and March 2005. Although 659 (1.5%) of serum samples were positive for SLEV, no WNV infection was detected in free-ranging birds collected in 2004. As early as January 2005, WNV was detected in a seroconversion so we suspect WNV was introduced before 2005 at the end of 2004 in all 5 sampling locations and in a variety of ecosystems: Córdoba, periurban (1.1%, 6/543); Mar Chiquita, thorn forest (5.1%, 16/313); Monte Alto, semidry chaco forest (9.8%, 8/82); Montecristo, cropland (9.5%, 2/21); and San Miguel de Tucumán, periurban yungas foothills (4.9%, 12/227).

**Table:** Prevalence of West Nile virus–neutralizing antibodies among birds grouped by taxonomic family, sampled in Chaco, Córdoba, and Tucumán Provinces, Argentina, 2004–2006*

Bird family	No. positive	No. tested	% Positive (95% CI)	Range of PRNT_80_ titer†
*Cardinalidae*	2	54	3.7 (1.0–12.5)	80–160
*Columbidae*	4	270	1.5 (0.6–3.8)	80–1,280
*Dendrocolaptidae*	4	17	23.5 (9.6–47.3)	320–2,560
*Falconidae*	3	5	60.0 (23.1–88.2)	320–2,560
*Furnariidae*	12	201	6.0 (3.4–10.1)	80–1,280
*Icteridae*	3	137	2.2 (0.7–6.2)	40–320
*Passeridae*	1	87	1.1 (0.2–6.2)	40
*Phasianidae*	2	8	25.0 (7.1–59.1)	320
*Polioptilidae*	2	7	28.6 (8.2–64.1)	80–640
*Troglodytidae*	1	17	5.9 (1.0–27.0)	80
*Turdidae*	8	132	6.1 (3.1–11.5)	40–1,280
*Tyrannidae*	1	370	0.3 (0.05–1.5)	160

*Most of these families are of the order Passeriformes except for *Falconidae* (Falconiformes), *Phasianidae* (Galliformes), and *Columbidae* (Columbiformes). CI, confidence internal, determined by the Wilson score method for binomial proportions, without continuity correction. †PRNT, plaque-reduction neutralization test. Titers are expressed as inverse of dilution.

In 2006, WNV was isolated from equines in Buenos Aires province (*5*). WNV transmission to resident birds collected further north in Córdoba, Chaco, and Tucumán provinces was detected in 2005 and 2006. Our data suggest that WNV was introduced into Argentina before 2005 and maintained naturally in enzootic foci where numerous bird species from many families were exposed. Presumably, as in North America, locally abundant passerine birds such as turdids (thrushes) are amplifying hosts. If common species of the *Furnariidae* (a family absent from temperate North America) prove to be competent hosts, they could play an important role in WNV transmission in Argentina because of their frequent exposure to WNV. Twelve (12.5%) of 96 *F. ruffus* sampled in 2005 and 2006 tested positive.

How WNV reached Argentina may never be known. Dispersal by migrating birds is a popular hypothesis, although relatively few North American breeding birds migrate to Argentina, and austral migrants number fewer than boreal migrants. Komar and Clark (*2*) suggested that bird species in the order Charadriiformes, such as shorebirds and terns, are candidates for carrying WNV from North America to South America due to long lasting high-level viremias, occasional persistent infectious viral loads in skin, and direct, long-distance flights. WNV spread southward from the United States to northern South America between 1999 and 2004 following a stepping stone pattern, consistent with spread by birds. Moreover, introduction of WNV into Argentina by migratory birds could explain the presence of the virus in many places in a brief period. However, for migratory birds (211 serum samples tested) in this study, serologic test results were negative.

The high titers of WNV-reactive antibody are strongly indicative of WNV infections. Overall, 216 serum specimens reacted by PRNT test against SLEV, WNV or both at titers ≥20. Sixty-eight serum samples remain unidentified. The large number of unidentified flavivirus-positive samples detected by PRNT, ELISA, or both (148/474) could be due to 1) false positives; 2) cross-reactions between WNV- and SLEV-reactive antibodies that prevented definitive diagnosis by PRNT; 3) cross-reactive antibody and multiple, heterologous flavivirus infections; 4) previous infections by both WNV and SLEV; and/or 5) presence of other flaviviruses circulating in Argentina. SLEV is endemic throughout Argentina and, like WNV, belongs to the Japanese encephalitis virus serocomplex. Hemagglutination-inhibiting antibodies against several Brazilian flaviviruses (e.g., Bussuquara, Ilheus, Rocio viruses) have been reported in the neotropical region of extreme northern Argentina (*7*), but these viruses have not been isolated in Argentina.

Our serologic data suggest that WNV has established itself in 4 ecologic regions in Argentina in a brief period. Additional studies are needed to define the reservoir hosts and vectors of WNV in Argentina, and most importantly, to define the public health risk this virus represents.
